# 

**Published:** 1911-05

**Authors:** 


### BOOKS RECEIVED

The Practical Medicine Series. Ten Volumes. Under the general editorial charge of Gustavus P. Head, M.D. Vol. I. General Medicine. Edited by Frank Billings, M.D. and J. H. Salisbury, M.D. Series 1911. Chicago: The Year Book Publishers. (Price, $1.50; entire series, $10.00.)

Annual Report of the Surgeon General of the Public Health and Marine-Hospital Service of the United States for the fiscal year 1910. Walter Wyman, M.D., Surgeon General.

Modern Diagnosis and Treatment of Diseases of Children. By Herman B. Sheffield, M.D., Instructor in Diseases of Children at the New York Post-Graduate Medical School and Hospital. Octavo, pp. 619. Illustrated. Philadelphia: F. A. Davis Co. 1911. (Cloth, $4.50.)

Diseases of the Stomach and Intestines. By Boardman Reed, M.D., Consulting Gastroenterologist to the Pottenger Sanatorium, Monrovia, Cal. Third edition. Octavo, pp. 1037. Illustrated. New York: E. B. Treat & Co. 1911. (Cloth, $5.00.)

Studies in the Psychology of Sex. Sex in relation to Society. Vol. VI. By Havelock Ellis. Octavo, pp. 656. Philadelphia: F. A. Davis Co. 1911. (Cloth, $3.00.)

International Clinics. A Quarterly of Illustrated Clinical Lectures and especially prepared articles on Treatment, Medicine, Surgery, Neurology, Pediatrics, Obstetrics, Gynecology, Orthopedics, Pathology, Dermatology, Ophthalmology, Otology, Rhinology, Laryngology, Hygiene, etc. Edited by Henry W. Cattell, M.D. Vol. I, twenty-first series. Philadelphia and London: J. B. Lippincott Co. 1910. (Cloth, $2.00.)

What Shall I Eat? A Manual of Rational Feeding. By Dr. F. X. Gouraud, Formerly Chief of the Laboratory of the Medical Faculty of Paris. With a Preface by Prof. Armand Gautier, of Paris. Only authorized translation by Francis J. Rebman. Octavo, pp. 379. New York: Rebman Co. 1911. (Cloth, $1.50.)

New and Nonofficial Remedies, 1911: Containing descriptions of articles which have been accepted by the Council on Pharmacy and Chemistry of the American Medical Association, prior to January 1, 1911. Pp. 282. (Paper, 25 cents; cloth, 50 cents.)

